# Supplementary material for: Modeling glioblastoma heterogeneity as a dynamic network of cell states
Source: Mol Syst Biol. 2021 Sep 16;17(9):e10105. doi: 10.15252/msb.202010105 (PMC8444284; doi:10.15252/msb.202010105)
Supplement: Supplementary file 5 — Source Data for Figure 3 [file MSB-17-e10105-s001.zip › Figure3A_sourcedata/GSEA_3065/nosigdb_state5.GseaPreranked.1622718435914/DOROSZKO_U3054MG_CLUSTER_0.html]

Details for gene set DOROSZKO\_U3054MG\_CLUSTER\_0[GSEA]

|  || Dataset | state5 |
| Phenotype | NoPhenotypeAvailable |
| Upregulated in class | na\_pos |
| GeneSet | DOROSZKO\_U3054MG\_CLUSTER\_0 |
| Enrichment Score (ES) | 0.5309499 |
| Normalized Enrichment Score (NES) | 1.8997546 |
| Nominal p-value | 0.0 |
| FDR q-value | 0.0039039073 |
| FWER p-Value | 0.03 |
Table: GSEA Results Summary

  

Fig 1: Enrichment plot: DOROSZKO\_U3054MG\_CLUSTER\_0      
 Profile of the Running ES Score & Positions of GeneSet Members on the Rank Ordered List

  

| PROBE | GENE SYMBOL | GENE\_TITLE | RANK IN GENE LIST | RANK METRIC SCORE | RUNNING ES | CORE ENRICHMENT || 1 | PTPRZ1 |  |  | 12 | 0.862 | 0.0551 | Yes |
| 2 | CD9 |  |  | 14 | 0.857 | 0.1111 | Yes |
| 3 | SOX6 |  |  | 28 | 0.754 | 0.1590 | Yes |
| 4 | NCAM1 |  |  | 70 | 0.581 | 0.1925 | Yes |
| 5 | TTYH1 |  |  | 141 | 0.489 | 0.2167 | Yes |
| 6 | COL9A3 |  |  | 144 | 0.489 | 0.2484 | Yes |
| 7 | MCAM |  |  | 174 | 0.460 | 0.2753 | Yes |
| 8 | SLC44A1 |  |  | 216 | 0.428 | 0.2987 | Yes |
| 9 | ALCAM |  |  | 225 | 0.422 | 0.3254 | Yes |
| 10 | CDH2 |  |  | 261 | 0.403 | 0.3479 | Yes |
| 11 | CADM1 |  |  | 307 | 0.378 | 0.3676 | Yes |
| 12 | CREB5 |  |  | 321 | 0.373 | 0.3905 | Yes |
| 13 | TMEM132B |  |  | 335 | 0.365 | 0.4130 | Yes |
| 14 | PRUNE2 |  |  | 344 | 0.363 | 0.4359 | Yes |
| 15 | TNFAIP6 |  |  | 380 | 0.349 | 0.4548 | Yes |
| 16 | ERBB3 |  |  | 411 | 0.337 | 0.4734 | Yes |
| 17 | BCHE |  |  | 425 | 0.333 | 0.4938 | Yes |
| 18 | CPM |  |  | 439 | 0.328 | 0.5138 | Yes |
| 19 | P2RX7 |  |  | 473 | 0.319 | 0.5309 | Yes |
| 20 | OLIG1 |  |  | 809 | 0.239 | 0.5091 | No |
| 21 | SNX22 |  |  | 1026 | 0.208 | 0.4985 | No |
| 22 | PCSK1N |  |  | 1162 | 0.190 | 0.4958 | No |
| 23 | COBL |  |  | 1186 | 0.187 | 0.5054 | No |
| 24 | SORL1 |  |  | 1247 | 0.180 | 0.5105 | No |
| 25 | SEMA6D |  |  | 1286 | 0.173 | 0.5176 | No |
| 26 | GPC4 |  |  | 1288 | 0.173 | 0.5288 | No |
| 27 | ADAM23 |  |  | 1586 | 0.142 | 0.5048 | No |
| 28 | FRZB |  |  | 1970 | 0.114 | 0.4694 | No |
| 29 | SLC6A8 |  |  | 2268 | 0.094 | 0.4422 | No |
| 30 | FIBIN |  |  | 2599 | 0.074 | 0.4101 | No |
| 31 | AZGP1 |  |  | 2661 | 0.070 | 0.4079 | No |
| 32 | MAPT |  |  | 4051 | 0.005 | 0.2526 | No |
| 33 | TRIM2 |  |  | 4188 | -0.001 | 0.2375 | No |
| 34 | SOX2 |  |  | 4271 | -0.005 | 0.2286 | No |
| 35 | PPP1R9A |  |  | 4335 | -0.007 | 0.2220 | No |
| 36 | AP002884.1 |  |  | 4524 | -0.015 | 0.2019 | No |
| 37 | SHC3 |  |  | 4768 | -0.025 | 0.1763 | No |
| 38 | FAM181B |  |  | 4789 | -0.025 | 0.1757 | No |
| 39 | UBXN2A |  |  | 5134 | -0.038 | 0.1397 | No |
| 40 | PDLIM3 |  |  | 5938 | -0.071 | 0.0544 | No |
| 41 | SFRP1 |  |  | 6042 | -0.076 | 0.0479 | No |
| 42 | ETS2 |  |  | 6076 | -0.078 | 0.0493 | No |
| 43 | TSC22D4 |  |  | 6599 | -0.108 | -0.0021 | No |
| 44 | MGLL |  |  | 7100 | -0.145 | -0.0485 | No |
| 45 | PPP2R2B |  |  | 7511 | -0.192 | -0.0819 | No |
| 46 | SLAIN1 |  |  | 7514 | -0.192 | -0.0695 | No |
| 47 | PPP1R18 |  |  | 7582 | -0.202 | -0.0638 | No |
| 48 | GLTP |  |  | 7609 | -0.205 | -0.0533 | No |
| 49 | PCBP4 |  |  | 7643 | -0.210 | -0.0433 | No |
| 50 | PMEPA1 |  |  | 7830 | -0.241 | -0.0484 | No |
| 51 | SAP30 |  |  | 7936 | -0.263 | -0.0429 | No |
| 52 | TUBB2B |  |  | 8224 | -0.332 | -0.0533 | No |
| 53 | CKB |  |  | 8448 | -0.407 | -0.0517 | No |
| 54 | KHDRBS3 |  |  | 8542 | -0.452 | -0.0325 | No |
| 55 | UCHL1 |  |  | 8700 | -0.565 | -0.0131 | No |
| 56 | FABP5 |  |  | 8829 | -0.684 | 0.0174 | No |
Table: GSEA details [plain text format]

  

Fig 2: DOROSZKO\_U3054MG\_CLUSTER\_0: Random ES distribution      
 Gene set null distribution of ES for **DOROSZKO\_U3054MG\_CLUSTER\_0**
